# Customizing AI‐based screening with real‐world data: Practical insights from diabetic retinopathy

**DOI:** 10.1111/aos.17591

**Published:** 2025-09-11

**Authors:** Broder Poschkamp, Liane Kantz, Petra Augstein, Allam Tayar, Lars Kaderali, Martin Busch, Beathe Bohl, Sebastian Paul, Lisa Lüdtke, Marie‐Christine Bründer, Daniel Schulz, Hanna Grabow, Elke Gens Dipl, Antonia Müller, Emily Martin, Wolfgang Kerner, Jörg Reindel, Andreas Stahl

**Affiliations:** ^1^ Department of Ophthalmology University Medicine Greifswald Greifswald Germany; ^2^ Department of Diabetology Heart‐ and Diabetes Centre Karlsburg Karlsburg Germany; ^3^ Leibniz Institute for Plasma Science and Technology Greifswald Germany; ^4^ Institute of Bioinformatics, University Medicine Greifswald Greifswald Germany

**Keywords:** AI adjustment, artificial intelligence, diabetic retinopathy, non‐mydriatic imaging, real world, Youden Index

## Abstract

**Purpose:**

Diabetic retinopathy (DR) is a leading cause of vision loss in middle‐aged adults globally. Although artificial intelligence (AI)‐based screening tools like IDx‐DR (classification) and Thirona RetCAD (regression) have shown high sensitivity in controlled settings, real‐world screening faces challenges due to missing or low‐quality images and inadequate adaptation to local healthcare needs. The objective was to compare the performance of two AI‐based DR screening algorithms (IDx‐DR and RetCAD) that analyse non‐mydriatic images, against ophthalmologists' mydriatic fundoscopy with image analysis and the impact of customized referral threshold modification (‘Greifswald modification’) on screening outcomes.

**Methods:**

This one‐centre observational study included 1716 patients with diabetes mellitus (Clinical Trials Register: DRKS00035967). Sensitivity, specificity, the proportion of ungradable images and the reduction in ophthalmologic evaluations were assessed. Customized referral threshold modification was conducted using the Youden Index.

**Results:**

In 98 patients (5.7%), no images could be acquired, and 35 patients (2.1%) had incomplete image sets for IDx‐DR. IDx‐DR rejected 438 patients (25.5%) due to image quality, while RetCAD flagged 134 eyes from 120 patients (6.9%) but provided output for all. Among analysable images, sensitivities ranged from 70.4% (RetCAD) to 93.6% (RetCAD with Greifswald modification). Including all patients reduced sensitivity from 52.7% (IDx‐DR) to 79.9% (RetCAD with Greifswald modification). AI screening reduced ophthalmologic exam needs by 47.5% to 78.5%.

**Conclusions:**

Real‐world DR screening performance of AI algorithms, when including non‐analysable patients, can be substantially lower than in controlled studies. The use of regression algorithms enabled the customization of referral thresholds, improving screening accuracy and reducing the clinical burden.

## 1 INTRODUCTION

The prevalence of diabetes mellitus is increasing globally (Sun et al., 2022). Approximately 35% of all diabetic patients develop diabetic retinopathy (DR), with one‐third of these individuals being at risk of vision loss (Lee et al., 2015). This makes DR the most common cause of blindness in working age adults in high‐income countries (Lightman & Towler, 2003). In recent years, various deep‐learning methods have been developed for DR screening (Bellemo et al., 2019; Grzybowski et al., 2020). Among them, IDx‐DR was the first autonomous system to receive FDA approval in 2018. It was validated in a multi‐centre clinical trial with 900 patients in a US population, comparing its performance to gold standard imaging and OCT grading (Abràmoff et al., 2018). RetCAD, developed by Thirona Retina (now distributed by iCare), is among more than 20 AI‐based ophthalmic algorithms currently available in the European Union and relies on convolutional neural networks trained on publicly available data sets (Messidor and Messidor‐2) and proprietary clinical images, and has been externally validated against expert graders on an image data set (Grzybowski & Brona, 2023; Grzybowski, Brona, et al., 2024; Thirona, 2020).

Many AI studies measure DR screening performance using high‐quality image datasets, overlooking real‐world challenges where images may be unavailable or of poor quality. This study assesses RetCAD and IDx‐DR for routine DR screening in a specialized diabetes centre, including patients without screening results. IDx‐DR classifies DR into discrete categories (no, mild, moderate or severe DR), while RetCAD provides a continuous severity score mapped to disease stages (González‐Gonzalo et al., 2020). Defining referral thresholds is crucial, with ‘more than mild’ DR often used internationally. This study compares algorithm sensitivity and specificity in real‐world screening and examines how a deep‐learning DR score from a regression algorithm can be tailored to specific populations.

## 2 METHODS

### 2.1 Study description

Patients with known diabetes mellitus who were treated at the Klinikum Karlsburg, a specialized centre for heart diseases and diabetes mellitus located in the Northeast of Germany, between February 2020 and December 2023 were included in this study upon signature of the informed consent form (in case of minors or patients with a legal representative, the informed consent was given by parents or the legal representative). Patients were predominantly of White European ethnicity, but collecting data on ethnic background is not routinely permitted in Germany due to historical and legal constraints. Ethics approval for this study was obtained from the Ethics Board at the University Medicine Greifswald (BB 025/20a; BB 025/20d) and the study was registered at the German Clinical Trials Register (DRKS00035967).

Trained non‐physician personnel recorded disease‐relevant data (e.g. age, sex, BMI, diabetes type/duration, insulin use, medication, prior ophthalmic surgery, HbA1c) and also examined diastolic/systolic blood pressure and visual acuity per eye. Fundus images were acquired using Topcon (TRC‐NW400 Topcon Deutschland GmbH, Willich, Germany) (97.7% of cases) or Zeiss Camera systems (Zeiss Visucam 500 Zeiss Gruppe, Oberkochen, Germany) (2.3% of cases). Obtained images were evaluated by the IDx‐DR algorithm (Digital Diagnostics Inc., Coralville, USA), the Thirona RetCAD algorithm v2.2.0 (Thirona retina, Nijmegen, Netherlands) and one of six ophthalmologists (independent from fundoscopy analysis). The IDx‐DR analysis was done continuously with patient inclusion; the RetCAD analyses were done retrospectively on the same image dataset.

An important aspect of this study was that no pharmacological pupil dilation was performed for the collection of fundus photographs. In some patients, no image could be acquired without pupil dilation (referred to as ‘no image’). The IDx‐DR algorithm has the following output: not analysable, no, mild, moderate or severe diabetic retinopathy (DR). The RetCAD algorithm uses a diabetic retinopathy score between 0 (no DR) and 5 (proliferative DR). For practical purposes, the output is mapped to five discrete stages by the manufacturer with the following DR scores and classes: 0–1: no DR, 1–2: mild DR, 2–3: moderate DR, 3–4: severe DR, 4–5: proliferative DR. In comparison to the IDx‐DR algorithm, the RetCAD algorithm analyses all images and provides a quality score ranging from 0 (lowest quality) to 100 (best quality). For images with a quality score below 25, the algorithm marks them as not analysable and suggests referral; however, it still provides a DR score in these cases, which can be used for further analysis.

As reference, all patients underwent funduscopic examination with pupil dilation by one of six experienced doctors with a mean of 13.5 ± 8.6 years' experience as ophthalmologists (range 5–31 years) within a 7‐day window after image acquisition. If a fundus image was available, the doctor could use it for diagnosis. This examination served as the gold standard for all comparisons. The ophthalmologist's grading was recorded according to ETDRS classification (Chew et al., 2010; Early Treatment Diabetic Retinopathy Study Research Group, 1991).

### 2.2 Screening metrics, data analysis and statistics

This analysis distinguishes sensitivity and specificity across all patients from those among diagnosable patients. The outcomes without screening result influence calculations, necessitating separate metrics (Figure S1 in the Supporting Information).

Data cleaning and formatting were carried out using Python 3.10 (Python Software Foundation) and Microsoft Excel (Microsoft Excel V.16.57, Redmond, WA, USA). Statistical analysis and data visualization were performed with Python 3.10 and GraphPad Prism (Version 10.3.0, USA).

Sensitivity, specificity and the receiver operating characteristic (ROC) analysis were conducted in Python. The Youden Index was computed for each DR stage to determine optimal screening thresholds (Youden, 1950). A Jupyter Notebook outlining the key steps of the analysis is included in the Supporting Information to support transparency and reproducibility.

## 3 RESULTS

### 3.1 Study characteristics

Between February 2020 and December 2023, a total of 1802 patients participated in the study. Of these, 11 patients declined permission for scientific use of their data after initial consent. Thus, the remaining cohort consisted of 1791 patients.

Out of this cohort, 75 patients could not undergo examination by an ophthalmologist for various reasons, such as being discharged before the medical exam, suspicion or confirmation of COVID‐19 infection, or failure to attend the scheduled appointment (referred to as ‘no exam’, Table S1 in the Supporting Information). This left 1716 patients who received a funduscopic examination with image analysis, which was considered the gold standard for this study.

The demographic characteristics of the study population are summarized in Table 1. For the analysis of diabetic retinopathy (DR) within the cohort, the diagnosis from the funduscopic examination was used, and the most severe DR classification from either eye was considered. Additional details, including age distribution (Figure S2), severity of DR and diabetes duration (Figure S3A,B), are provided in the supplement.

**TABLE 1:** Study demographics.

	All with exam	Diabetic retinopathy severity in worse eye (*n* = 1716)			
		No	Mild	Moderate	Severe DR
Patients, No.	1716	1046	357	179	134
Female, No. (%)	729 (42.5%)	600 (57.4%)	155 (43.4%)	82 (45.8%)	46 (34.3%)
Male, No. (%)	987 (57.5%)	446 (42.6%)	202 (56.6%)	97 (54.2%)	88 (65.7%)
*Diabetes type*					
Type 1, No. (%)	885 (51.6%)	493 (47.1%)	190 (53.2%)	112 (62.6%)	90 (67.2%)
Type 2, No. (%)	763 (44.5%)	497 (47.5%)	158 (44.3%)	66 (36.9%)	42 (31.3%)
Other types, No. (%)	21 (1.2%)	14 (1.4%)	6 (1.7%)	0	1 (0.7%)
Unknown, No. (%)	47 (2.7%)	42 (4.0%)	3 (0.8%)	1 (0.5%)	1 (0.7%)
*Age*					
Years (mean ± SD)	50.4 ± 18.5	47.2 ± 20.0	54.7 ± 14.7	57.7 ± 12.8	54.5 ± 15.0
Age range (min–max)	6–97	6–92	13–97	18–86	14–85
*Diabetes duration*					
Years (mean ± SD)	16.8 ± 13.8	11.2 ± 10.3	23.1 ± 12.9	28.2 ± 13.9	29.9 ± 14.6
*HbA1c*					
% (mean ± SD)	8.8 ± 2.1	8.9 ± 2.1	8.6 ± 2.0	8.5 ± 1.5	8.9 ± 2.0
*BMI*					
kg/m^2^ (mean ± SD)	29.9 ± 7.4	29.7 ± 7.7	31.0 ± 7.3	29.8 ± 6.0	29.4 ± 5.5

### 3.2 Performance of the IDx‐DR and RetCAD algorithm for diagnosable patients

**FIGURE 1:**
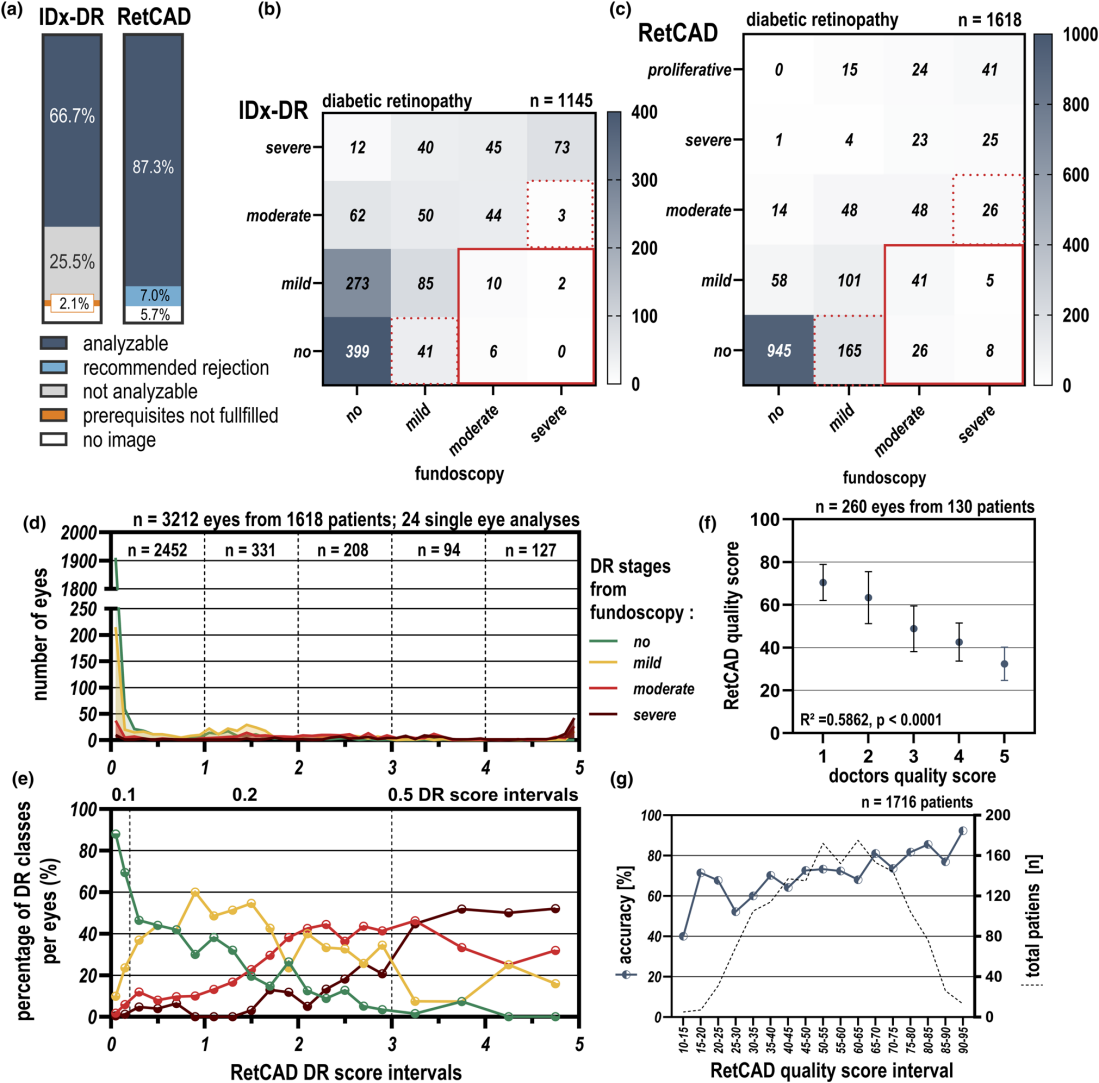
Analysis of IDx‐DR and RetCAD algorithm performance. (a) The percentage of analysable, non‐analysable and unavailable images for the IDx‐DR and RetCAD algorithms is illustrated. The IDx‐DR algorithm could analyse 66.7% of cases (*n* = 1145), while 25.5% were not analysable due to poor image quality, and 7.8% lacked the necessary number of images. In contrast, the RetCAD algorithm was able to analyse 94.3% of cases (*n* = 1618 including 134 eyes from 120 patients analysable but recommended for rejection due to poor quality), with 5.7% having no available images. (b) A confusion matrix comparing the IDx‐DR algorithm's performance (*n* = 1145) against the doctor's fundoscopic diagnosis comparing the following diabetic retinopathy (DR) stages: No DR, mild, moderate and severe DR. The red dotted lines highlight instances where the algorithm underestimated the severity compared to the fundoscopic diagnosis, with the red solid box indicating cases where more than mild DR was missed (*n* = 18). (c) The confusion matrix for the RetCAD algorithm (*n* = 1618) similarly compares the algorithm's grading against the gold standard of fundoscopic diagnosis. This matrix includes an additional category of proliferative DR. The red dotted lines highlight instances where the algorithm underestimated the severity compared to the fundoscopic diagnosis, with the red solid box indicating cases where more than mild DR was missed (*n* = 80). (d) The distribution of eyes (*n* = 3212) from 1618 patients (including 24 single‐eye analyses) across various RetCAD DR scores is illustrated. The number of eyes for each score is displayed, with the majority of eyes (*n* = 2452) scoring between 0 and 1. The coloured lines represent the different DR stages as diagnosed by fundoscopy: Green for no DR, yellow for mild DR, red for moderate DR and brown for severe DR. (e) The percentage of eyes in each DR stage relative to their Thirona DR score is shown. The DR scores are categorized into intervals: 0 to 0.2 (in 0.1 intervals), 0.2 to 3.0 (in 0.2 intervals) and 3.0 to 5.0 (in 0.5 intervals). For each RetCAD DR score interval, the y‐axis represents the percentage of eyes in each DR stage. (f) This section presents the distribution of DR scores across different categories of image quality, as rated by the ophthalmologist. Each category of image quality is represented by individual values, with horizontal bars indicating the mean and standard deviation for each group. (g) The relationship between the RetCAD algorithm's image quality score (*x*‐axis) and its accuracy in detecting diabetic retinopathy (left *y*‐axis) is displayed. The quality scores are grouped into intervals, with each point representing the accuracy for that interval. The algorithm achieved increasing accuracy with higher quality scores. The number of patients per quality score is presented (right *y*‐axis).

A total of 1716 patients underwent fundoscopic examination, of which image acquisition was unsuccessful for 98 patients (5.7%). For the IDx‐DR analysis, an additional 35 patients (2.1%) failed to provide the required four images, two macula‐centred and two optic disc‐centred, resulting in a total of 133 patients (7.8%) lacking the necessary imaging prerequisites. Additionally, in 438 patients (25.5%), the images were not analysable by the IDx‐DR algorithm. In cases of inadequate image quality, the IDx‐DR algorithm does not provide any feedback regarding the diabetic retinopathy (DR) stage. Consequently, 1145 patients (66.7%) were eligible for IDx‐DR analysis (Figure 1a). In contrast, the RetCAD algorithm was applied to all patients with available images (*n* = 1618, which corresponds to 94.3%, including 24 patients with images from only one eye, Figure 1a). The RetCAD algorithm recommended the exclusion of 134 eyes from 120 patients (6.99%) from analysis due to image quality; nevertheless, it provided an output score also for these patients. In this cohort, 14 patients had both eyes recommended for exclusion by the RetCAD algorithm.

The diagnostic sensitivity of the IDx‐DR algorithm for detecting more than mild DR on analysable images was 90.16%, while the sensitivity for severe DR was 93.58%. The diagnostic specificity for distinguishing between no DR and mild DR was 82.95% (Figure 1b). The diagnostic sensitivity of the RetCAD algorithm for detecting more than mild DR on analysable images (including the ones recommended to be rejected) was 70.41%, and the sensitivity for severe DR was 62.86%. The specificity for no DR and mild DR was 93.86% (Figure 1c). When comparing individual classifications, the RetCAD algorithm shows strong performance in identifying cases without DR (high specificity) but demonstrates lower sensitivity in detecting moderate and severe DR compared to the IDx‐DR algorithm.

The RetCAD algorithm results in a DR score which was further investigated. A total of 3212 eyes from 1618 patients were analysed, including 24 assessments where only one eye from a patient was analysable. In our population, 2172 outputs (67.6%) fell within the RetCAD DR score range from 0 to 0.1 (Figure 1d). To analyse funduscopic diagnoses across DR scores, RetCAD scores were grouped into intervals. As DR scores increased, moderate and severe DR rose, while no DR declined. Mild DR remained stable at lower to mid‐range scores but decreased at higher intervals (Figure 1e).

To investigate the Thirona quality score, an ophthalmologist assessed image quality for 260 eyes from 130 patients, rating it on a scale from 1 (best quality) to 5 (uninterpretable). The ophthalmologist's assessment was compared with the RetCAD quality score, showing a significant linear regression between the two quality scores (*R* ^2^ = 0.586, *p* < 0.0001, *F*‐Test). Images rated as ‘uninterpretable’ by the ophthalmologist (score of 5) had a corresponding RetCAD quality score of 32.38, while images of the highest quality (score of 1) had a RetCAD quality score of 70.43 (Figure 1f).

The accuracy of the RetCAD algorithm is dependent on the image quality score, but it is notable that, for a quality score above 30, the algorithm consistently achieved accuracy over 60.0%. Only four patients in the study presented images with a quality score below 15 (Figure 1g). There was no significant correlation between image quality and DR score variability; however, once the DR score exceeded 3.5, the standard deviation increased, suggesting that higher DR scores may require additional images for reliable classification (Figure S4).

### 3.3 Adjustment of the RetCAD algorithm for the study population

**FIGURE 2:**
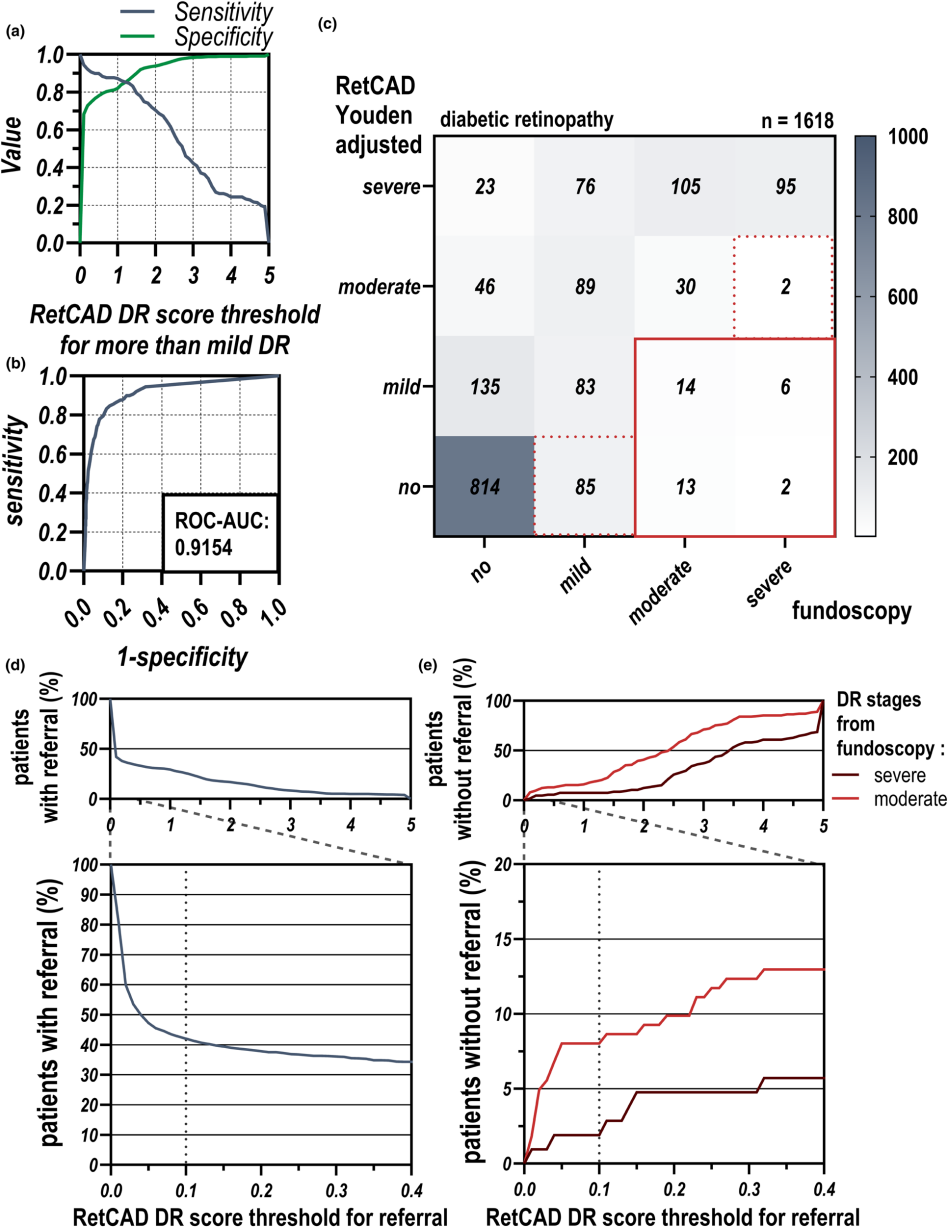
Performance and threshold analysis of the RetCAD algorithm for detecting more than mild DR. (a) The sensitivity (blue line) and specificity (green line) of the RetCAD algorithm are plotted as a function of the DR score threshold for detecting more than mild DR. With increasing DR score threshold, sensitivity decreased while specificity increased. The intersection of these curves indicates a potential optimal threshold for referral (Youden Index). (b) The receiver operating characteristic (ROC) curve for detecting more than mild DR is displayed, with an area under the curve (AUC) of 0.9154 demonstrating strong diagnostic performance. (c) A confusion matrix comparing the RetCAD algorithm (adjusted for the DR score threshold) against the gold standard fundoscopic diagnosis for 1618 patients. The matrix categorizes patients by DR severity (no DR, mild, moderate and severe), showing agreement and discrepancies between the algorithm's predictions and the clinical diagnosis. The red box highlights missed cases of moderate and severe DR (*n* = 35), while the red dotted lines indicate areas of underestimation. (d) The percentage of patients requiring referral is shown across various DR score thresholds. Lower thresholds resulted in a higher referral rate, while higher thresholds reduced the number of patients referred for further evaluation. (e) The percentage of patients with moderate (red line) and severe DR (brown line) who would be not referred at different DR score thresholds is displayed. As the threshold increased, the number of missed moderate and severe DR cases rose.

Unlike IDx‐DR, RetCAD generates a continuous DR score, enabling adjustments for specific populations. Based on diagnosable patients, the sensitivity and specificity of the RetCAD algorithm were calculated by determining a potential DR score threshold indicating more than mild DR (Figure 2a). Additionally, a receiver operating characteristic (ROC) curve was generated to assess the performance for detecting more than mild DR, yielding an area under the curve (AUC) of 0.9153 (Figure 2b).

To establish optimal thresholds for different stages of diabetic retinopathy (DR), we employed ROC curve analysis and the Youden index. This analysis identified the following thresholds for DR stages: no DR (0–0.07), mild DR (>0.07–1.02), moderate DR (>1.02–1.67) and severe DR (>1.67). We displayed the changed screening situation (Figure 2c).

The RetCAD DR score close to 0 is of particular interest, as 67.6% of all patients fell within a score from 0 to 0.1. Using a potential referral threshold for the RetCAD DR score, the proportion of patients was visualized (Figure 2d). Additionally, we illustrate the proportion of patients with severe and moderate DR that would not be detected by the algorithm at this threshold (Figure 2e).

Based on this analysis, we recommend a referral threshold at a RetCAD DR score of >0.1 to maximize sensitivity for detecting moderate and severe DR. It is important to note that this threshold was developed on our screening population of patients with diabetes mellitus in Greifswald (Germany) and may differ from ideal thresholds in other screening populations. For our screening population and using the thresholds described above, the sensitivity of the modified RetCAD algorithm for detecting more than mild DR was 93.63%, and the specificity for identifying no or mild DR was 66.5%. Under this scenario, 2 severe DR cases and 13 moderate DR cases would be missed compared to 13 severe DR and 67 moderate DR cases with unmodified RetCAD screening.

### 3.4 Screening performance in all versus diagnosable patients

This section provides a comparison of the sensitivities and specificities of screening algorithms, both for patients with results from the algorithm and for all patients with fundoscopy results (Table 2).

**TABLE 2:** Screening characteristics compared to fundoscopy.

		Ophthalmologist on image	IDx‐DR	RetCAD	RetCAD Youden adjusted	RetCAD Greifswald modification^a^
Diagnosable	*Sensitivity*_*d*_					
	Patients, No.	1217	1145	1618	1618	1618
	more than mild DR/Referable DR	78.23%	90.16%	70.41%	86.89%	93.63%
	severe DR	77.11%	93.58%	62.86%	90.47%	97.14%
	*Specificity*_*d*_					
	no DR or mild DR	99.41%	82.95%	93.86%	82.67%	69.06%
All	*Sensitivity*_*a*_					
	Patients, No.	1716	1716	1716	1716	1716
	More than mild DR/Referable DR	48.24%	52.72%	60.06%	74.14%	79.87%
	Severe DR	47.76%	54.48%	49.25%	70.90%	76.12%
	*Specificity*_*a*_					
	No DR or mild DR	72.56%	56.88%	90.38%	79.62%	66.5%
Screening^b^	Patients needing referral, No.	656	900	369	564	766
	% less ophthalmologist exams	−61.7%	−47.55%	−78.49%	−67.13%	−55.36%
	Severe DR missed, No.	4	2	13	8	2

^a^ In the modified Greifswald version of the RetCAD algorithm, every patient with a RetCAD DR score above 0.1 is referred to an ophthalmologist. The intervals for DR classification (no DR, mild DR, moderate DR, severe DR) remain consistent with the RetCAD algorithm as adjusted by the Youden index.

^b^ The presented values are based on a hypothetical screening scenario with subsequent ophthalmologist follow‐up of all patients with no image, not analysable, more than mild DR or referable DR.

To validate the performance of the algorithms, images were evaluated by an ophthalmologist. Of the total cohort, 1217 patients (70.92%) had analysable images according to the ophthalmologist. The number of analysable images ranged from 1145 (IDx‐DR) to 1618 (RetCAD algorithm).

Among the algorithms compared, the RetCAD algorithm with the Greifswald modification demonstrated the highest sensitivity. It achieved a sensitivity of 93.63% for detecting ‘more than mild’ diabetic retinopathy (DR) in diagnosable images, and 79.87% sensitivity across the entire patient population. However, this increase in sensitivity was accompanied by a reduction in specificity (66.5%). Overall, the Greifswald modification to the RetCAD algorithm resulted in a 55.36% reduction in the number of required ophthalmological examinations, which is comparable to an ophthalmologist screening on images, which lead to a reduction of 61.7%.

The highest specificity over the entire population was achieved with the RetCAD algorithm without modifications, which was 90.38%. However, the sensitivity for detecting more than mild DR in patients with images was low (60.06%).

The highest sensitivity for detecting severe DR was achieved by the RetCAD algorithm with Greifswald modification on patients with images (97.14%) and over the entire population (76.12%). The number of patients with undetected severe DR is critical in all screening contexts as it represents those individuals who are not referred due to being falsely classified as having only mild or moderate DR. It is therefore important to have high sensitivity in screening algorithms along with a low number of eyes yielding ‘not analysable’ or ‘no image’ as a screening output.

## 4 DISCUSSION

This study demonstrated that adjusting AI‐based regression algorithms with real‐world data significantly improves sensitivity while still reducing clinical burden. It also revealed a notable gap between outcomes in controlled settings and the real world, where all patients must be considered. This example of algorithm adaptation offers valuable insights for AI algorithm development, real‐world evaluation and the global implementation of AI‐assisted diabetic retinopathy screening (Bellemo et al., 2019; Grzybowski, Brona, et al., 2024).

### 4.1 Image acquisition challenges and algorithmic robustness

A significant challenge in real‐world DR screening is the acquisition of high‐quality images without pupil dilation. In our study, 5.7% or 7.8% of patients could not be analysed with the RetCAD or IDx‐DR algorithm, respectively, due to missing images. The IDx‐DR algorithm requires two images per eye from each patient, which can be problematic for patients with only one eye or incomplete image sets. In contrast, the RetCAD algorithm is more flexible, accepting one to eight images per eye and allowing for analysis of single eyes. Although providing additional images may theoretically enhance algorithm sensitivity, standardized protocols focusing on high‐quality macula‐ and disc‐centred images offer a good balance between image acquisition time and reliable performance in real‐world settings (Goh et al., 2016).

In our study, the analysis of the IDx‐DR algorithm was rejected for 25.5% of all patients. The high number of not analysable images can be explained by non‐mydriatic pupils. This aligns with findings from other studies where the percentage of non‐analysable images from human image grading ranged between 2.47% (with pupil dilation) and 16.23% (only pupil dilation, where images could not be captured in miosis) (Lee et al., 2021). Given the high variability in imaging conditions in real‐world settings, particularly in low‐resource environments, AI algorithms must be robust to these challenges (Ruamviboonsuk et al., 2022; Wintergerst et al., 2020).

Generalizability remains a key challenge for AI‐based diagnostic systems, particularly when applied beyond their intended use or across varying imaging devices or when including a broader patient population than defined in the CE‐conformity statements of the evaluated algorithms, for example, paediatric patients. Ethnicity is also known to influence algorithm performance (Ting et al., 2017); however, our population was predominantly White European, and due to legal constraints in Germany, we were unable to assess performance across ethnic subgroups. Furthermore, ethical considerations around autonomous decision‐making, along with the shortage of longitudinal randomized controlled trials comparing AI outputs to ophthalmologists, continue to pose significant barriers to widespread clinical adoption (Ong et al., 2025).

### 4.2 Sensitivity and specificity of DR screening algorithms

A key observation from this study is the discrepancy between the high sensitivity and specificity values reported by DR screening algorithms and their performance in real‐world settings without excluding missing patients or images (Grzybowski et al., 2020). In many reported cases, only patients with images and images of good quality were included for analyses of sensitivity and specificity (Bellemo et al., 2019; Grzybowski, Jin, et al., 2024). The initial evaluation study of IDx‐DR included 892 patients who completed all procedures, and 73 patients of them could not be fully analysed. The sensitivity for more than mild DR on this data set was 87.2%, which is comparable to our result of diagnosable patients (90.2%), but not for all patients in our study (52.7%) (Abràmoff et al., 2018).

A continuous DR severity score, originated from a regression algorithm, allows for the adjustment of screening thresholds tailored to a specific population (Poschkamp & Stahl, 2022). This is important because AI algorithms can perform inconsistently in different patient populations (Ting et al., 2017). Sensitivity and specificity can vary dramatically depending on the defined threshold of the DR score and determining these values depends on the population and the specific needs of the screening programme. Additionally, differences arise based on the stage of diabetic retinopathy (e.g. mild, moderate, severe, more than mild, proliferative) and the classification of the condition as referable or non‐referable DR, which are not equivalent. In our approach, the cut‐offs between different DR stages were determined using the Youden Index (Fluss et al., 2005). The threshold for referable DR was set manually, taking available resources, technological capabilities and the acceptable level of risk for missed cases into account. For example, in countries with lower overall health care resources, the threshold for referable DR can be set for severe DR, proliferative DR or diabetic macular oedema instead of more than mild DR (Ruamviboonsuk et al., 2022). In our study, the threshold was tailored towards our specific screening population, leading to the name ‘Greifswald modification’. Given that only a small proportion of AI‐based medical devices have undergone head‐to‐head comparisons in studies with other AI systems (8%, 10/131) or human experts (22%, 29/131) (Ong et al., 2025), we emphasize the importance of population‐specific calibration as a preparatory step for clinical integration and large‐scale roll‐out. Threshold optimization using tools such as the Youden Index should be conducted once during initial validation on representative samples, rather than applied as an ongoing adjustment during routine use.

### 4.3 Uncertainty

For the assessment of image quality, we conducted a subjective 1–5 quality rating and we acknowledge that this was not based on a standardized or validated scale. Image quality can be assessed more objectively using structured approaches, such as a three‐domain system evaluating location, clarity and artefacts, each graded on a three‐level scale to derive an overall quality score (Guo et al., 2024). Adopting such standardized frameworks in future studies could improve the objectivity and comparability of image quality assessments.

Beyond this, algorithms should provide diagnostic certainty in addition to image quality (Poschkamp & Stahl, 2022). Currently, image quality only determines whether a DR diagnosis should be made, offering no insight into result reliability. A more informative approach would pair a DR score with an indication of how image quality affects diagnostic accuracy. Furthermore, it is important to indicate and, if feasible, quantify which specific retinal lesions (e.g. microaneurysms, haemorrhages, exudates) contribute to the final DR score assigned by the algorithm, as well as which lesions are commonly missed (Similié et al., 2025). The DR score is not interval scaled. Therefore, shifts in severity may not uniformly reflect the same disease progression. Still, a continuous DR score enables finer distinctions between disease stages, which better reflect the biological nature of disease progression. In other diseases, such as retinopathy of prematurity, the severity of disease stages has been shown to be classified differently by human specialists (Smith et al., 2019). AI‐based scores could also help to objectify disease stage classification (Campbell et al., 2021; Eilts et al., 2023).

### 4.4 Embedding DR algorithms in a screening process with local healthcare needs

The screening process should be embedded within a well‐defined screening scenario to clarify how the potential screening will be conducted, as demonstrated in the British or Singaporean screening programmes (Bellemo et al., 2019; Heydon et al., 2021; Nguyen et al., 2016). The screening model we used is a so‐called fully automated DR screening system, in which the algorithm determines whether a patient should be referred to an eye clinic. To enhance specificity, secondary graders could be included for cases identified as referable.

In Germany, current practice involves that diabetic patients undergo fundoscopic examination by an ophthalmologist every 2 years or annually in the presence of comorbidities (Arzneimittelkommission der deutschen Ärzteschaft et al., 2015). When embedding our screening approach, it is crucial to account for patients who might be missed, such as those with no images or non‐analysable images, as well as the 75 patients in our study who were available for AI‐based screening but did not undergo the recommended fundoscopy. A key finding of our study is the substantial potential of AI‐based DR screening algorithms to reduce the number of required ophthalmological exams by 47.5% to 78.5%, depending on how the screening process is implemented.

Our analysis of ophthalmologist's evaluations on fundus images suggests that current algorithms have achieved a high level of accuracy, potentially nearing the maximum achievable performance with the existing technology. Further advancements in screening efficacy could be realized by developing or using imaging technologies with an expanded field of view or by designing cameras that are more reliable and user‐friendly for capturing images from non‐mydriatic pupils (Talks et al., 2015). These improvements would not only enhance the diagnostic capabilities of AI algorithms but also increase the inclusiveness and efficiency of screening programmes by reducing the number of non‐analysable images.

## FUNDING INFORMATION

This work was funded by intramural grants at the Department of Ophthalmology, University Medicine Greifswald and Klinikum Karlsburg. Broder Poschkamp received individual funding from the Deutsche Forschungsgemeinschaft (DFG, German Research Foundation)—Project Number 493623784 and Gerhard Domagk Nachwuchsförderprogramm (Clinician Scientist Program Rural_Age Scholarship).

## CONFLICT OF INTEREST STATEMENT

The study was supported by complimentary AI‐based analyses of 1791 patients, made possible by Thirona Retina. The findings and interpretations presented in this study remain solely under the responsibility of the authors, without any external influence from Thirona Retina or other parties. B.P., Li.K, P.A., A.T., La.K., B.B., S.P., L.L., D.S., H.G., E.G., A.M., E.M., W.K., J.R., No conflict of interest to declare. M.Bu., Speaker: Bayer; Research grants: Bayer, Roche. M.Br., Scientific advisory board: abbvie. A.S.: Speaker: Astellas, Bayer, Novartis, Roche; Scientific advisory boards: Alcon, Apellis, Bayer, Novartis, Roche; Research grants: Bayer, Novartis, Roche; Clinical trials: Bayer, Novartis, Roche; Board of Directors: SemaThera Inc.

## Supporting information


Data S1.
