# Supplementary material for: Customizing AI‐based screening with real‐world data: Practical insights from diabetic retinopathy
Source: Acta Ophthalmol. 2025 Sep 11;104(3):323–32. doi: 10.1111/aos.17591 (PMC13058676; doi:10.1111/aos.17591)
Supplement: Supplementary file 1 — Data S1. [file AOS-104-323-s001.zip › aos17591-sup-0002-Supinfo2@supplement_V1.docx]

# **Supplement**

## **Screening metrics for image-based screening**

In a screening, each patient is classified either as disease-negative (e.g. no DR, test negative) or disease-positive (e.g., presence of DR, test positive). The outcome of a screening test is compared to a gold standard, typically a physician’s examination with the results of a present disease (disease positive, DP) or without a disease (disease negative, DN). AI-based screening introduces additional categories like “no image” or “not analyzable,” grouped as “no result” (NR), as these cases cannot be classified reliably.

This analysis distinguishes two sensitivity metrics: sensitivity_a_ across all patients and sensitivity_d_ among diagnosable patients. Similarly, specificity is differentiated as follows: specificity_a_ across all patients and specificity_d_ among diagnoseable patients. This differentiation is necessary because the “no result” category affects the calculation of screening performances.

The formulas for these metrics are as follows (**eFigure 1A**):

- Sensitivity for diagnosable patients (sensitivity_d_):

$$\mathrm{sensitivit}y_{d}=\frac{\mathrm{TP}}{TP+FN}$$

- Sensitivity for all patients (sensitivityₐ):

$$\mathrm{sensitivit}y_{a}=\frac{\mathrm{TP}}{TP+FN+DP\text{-}\mathrm{NR}}$$

- Specificity for diagnosable patients (specificity_d_):

$$\mathrm{specificit}y_{d}=\frac{\mathrm{TN}}{TN+FP}$$

- Specificity for all patients (specificityₐ):

$$\mathrm{Specificit}y_{a}=\frac{\mathrm{TN}}{TN+FP+DN\text{-}\mathrm{NR}}$$

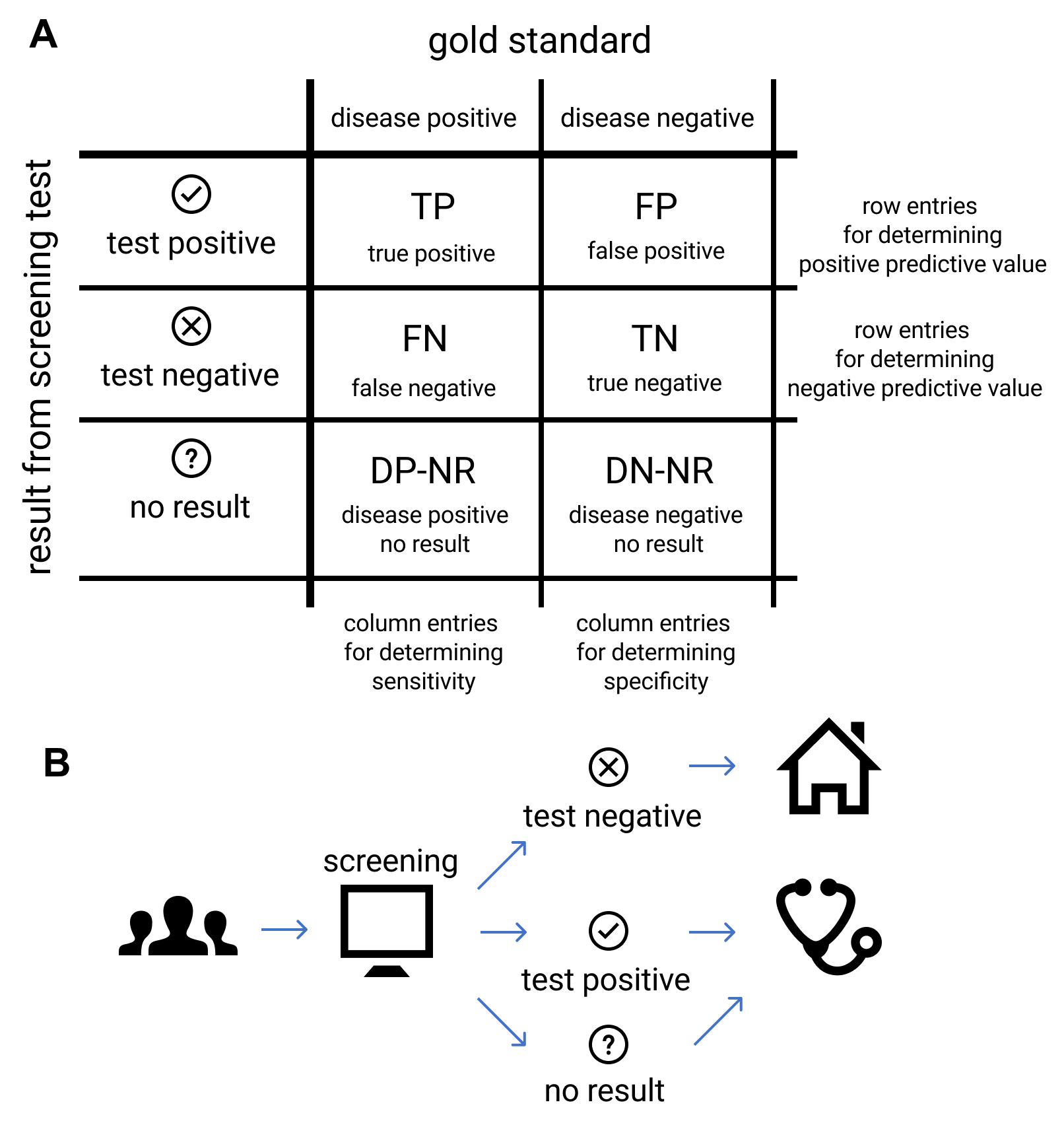


**eFigure 1: Image-based screening situation**

**A:** The contingency table represents the outcomes of an image-based screening scenario. Notably, there is the possibility that no test result is available, i.e. some individuals might not have received a definitive diagnosis. This necessitates different calculation methods for sensitivity and specificity, as indicated for groups DP‑NR and DN‑NR.

**B:** This section outlines the steps in a potential AI-based screening approach. Initially, the target population (i.e. individuals with diabetes) undergoes analysis through a screening algorithm. Those identified as positive by the algorithm are referred to a doctor for further examination, while individuals classified as negative are not examined further. However, individuals for whom the AI system yields no result must also be referred for a doctor's evaluation.

## **Additional Study demographics**

For additional information we present demographic information on our study population below.

**eTable 1: Study demographics for all patients and patients without fundoscopy examinations**

|  | **All** | **exam** | **No exam** |
| --- | --- | --- | --- |
| **sex** |  |  |  |
| patients, No. | 1791 | 1716 | 75 |
| female No. (%) | 759 (42.4 %) | 729 (42.5 %) | 30 (40 %) |
| male No. (%) | 1032 (57.6 %) | 987 (57.5 %) | 45 (60 %) |
| **diabetes type** |  |  |  |
| type 1, No. (%) | 928 (51.8 %) | 885 (51.6 %) | 43 (57.3 %) |
| type 2, No. (%) | 791 (44.2 %) | 763 (44.5 %) | 28 (37.3 %) |
| other type, No. (%) | 24 (1.3 %) | 21 (1.2 %) | 2 (2.6 %) |
| Unknown | 48 (2.7 %) | 47 (2.7 %) | 1 (1.3 %) |
| **age** |  |  |  |
| years (mean ± SD) | 50.2 ± 18.5 | 50.4 ± 18.5 | 48.7 ± 19.6 |
| Age range (min-max) | 6-97 | 6-97 | 8-82 |
| **diabetes duration** |  |  |  |
| years (mean ± SD) | 16.9 ± 13.8 | 16.8 ± 13.8 | 16.5 ± 13.5 |
| **HbA1c** |  |  |  |
| % (mean ± SD) | 8.8 ± 2.1 | 8.8 ± 2.1 | 9.4 ± 2.2 |
| **BMI** |  |  |  |
| kg/m² (mean ± SD) | 30.0 ± 7.4 | 29.9 ± 7.4 | - 1. ± 9.2 |

## **Age distribution**


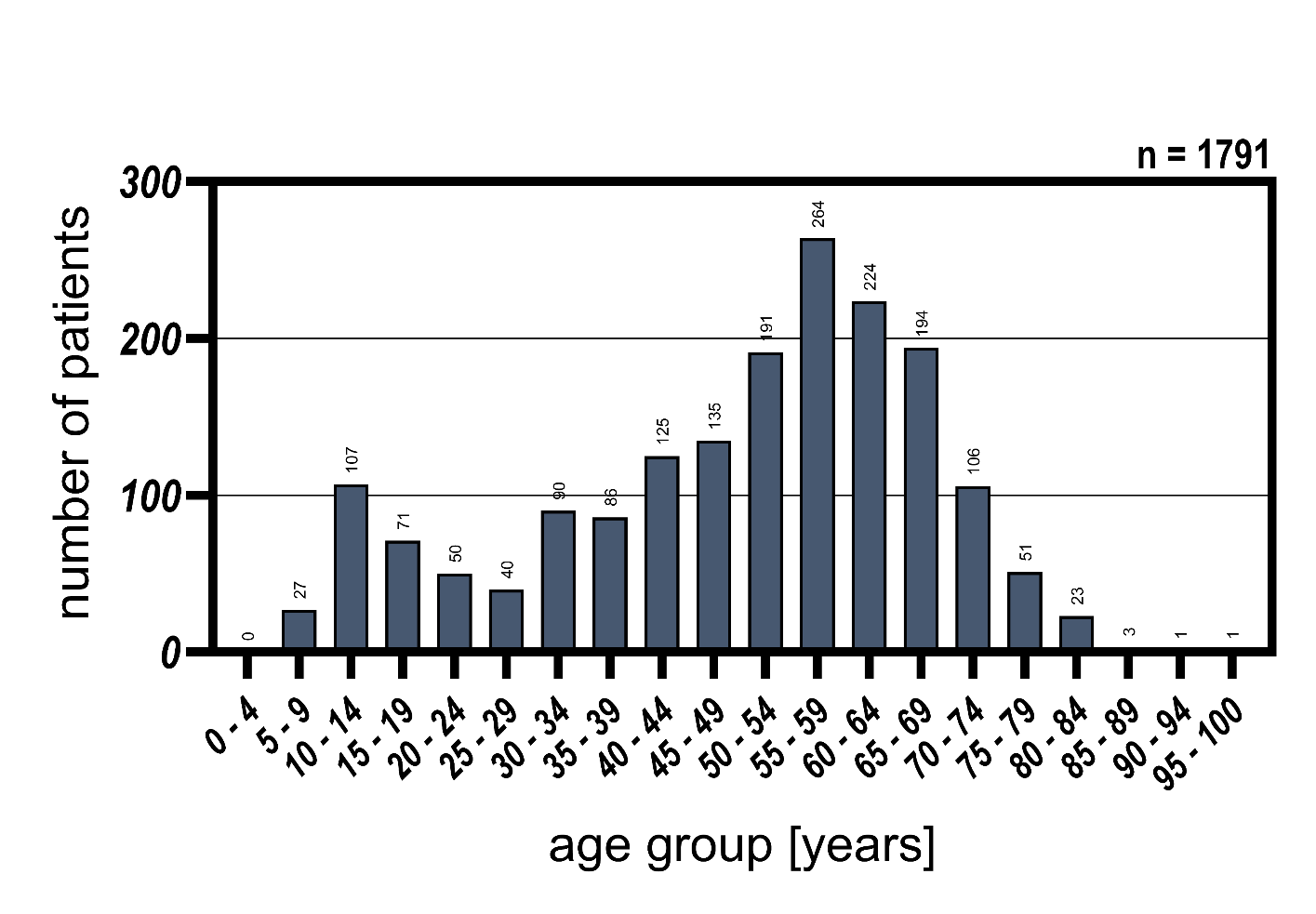
The mean age was 50.3 years, with a range spanning from 6 to 97 years. The interquartile range (IQR) of 24 years reflects that 50 % of the patients fell between the ages of 40 and 64 years, highlighting a middle-aged population as the majority. The median age was 55 years (**eFigure 2**).

**eFigure 2: age distribution of the study cohort**

## **Severity of diabetic retinopathy, age, and duration of diabetes**

For further information, the stages of diabetic retinopathy are presented in relation to the age of the patients as percentage of all patients with diabetes from funduscopic examination. We also present how many patients did not attend a follow-up examination by an ophthalmologist (**eFigure 3 A**).


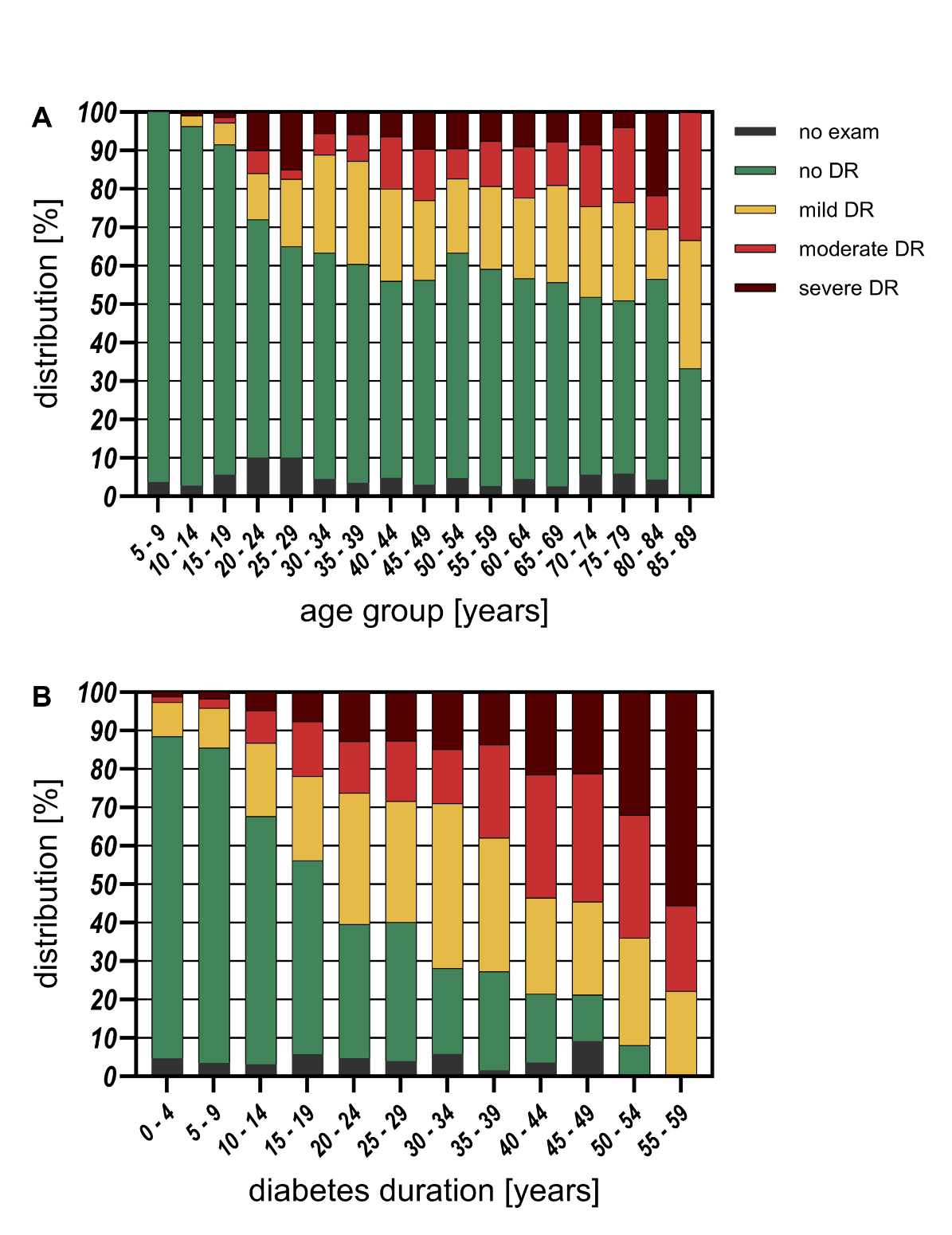
Additionally, disease severity is shown in relation to the duration of diabetes for all diabetic patients (**eFigure 3 B**).

**eFigure 3: Disease severity of diabetic retinopathy in relation to age and duration of diabetes**

## **RetCAD Quality Score and DR Score Standard Deviation**

The relationship between image quality and DR score variability was investigated to determine if lower image quality leads to greater fluctuations in DR scores. Across all eyes, no significant correlation (R² = 0.0005; p = 0.19) between DR score fluctuations and the RetCAD quality score was found. This suggests that the algorithm maintains its robustness even with lower-quality images, minimizing the impact of image quality on DR score variability (**eFigure 4 A**).


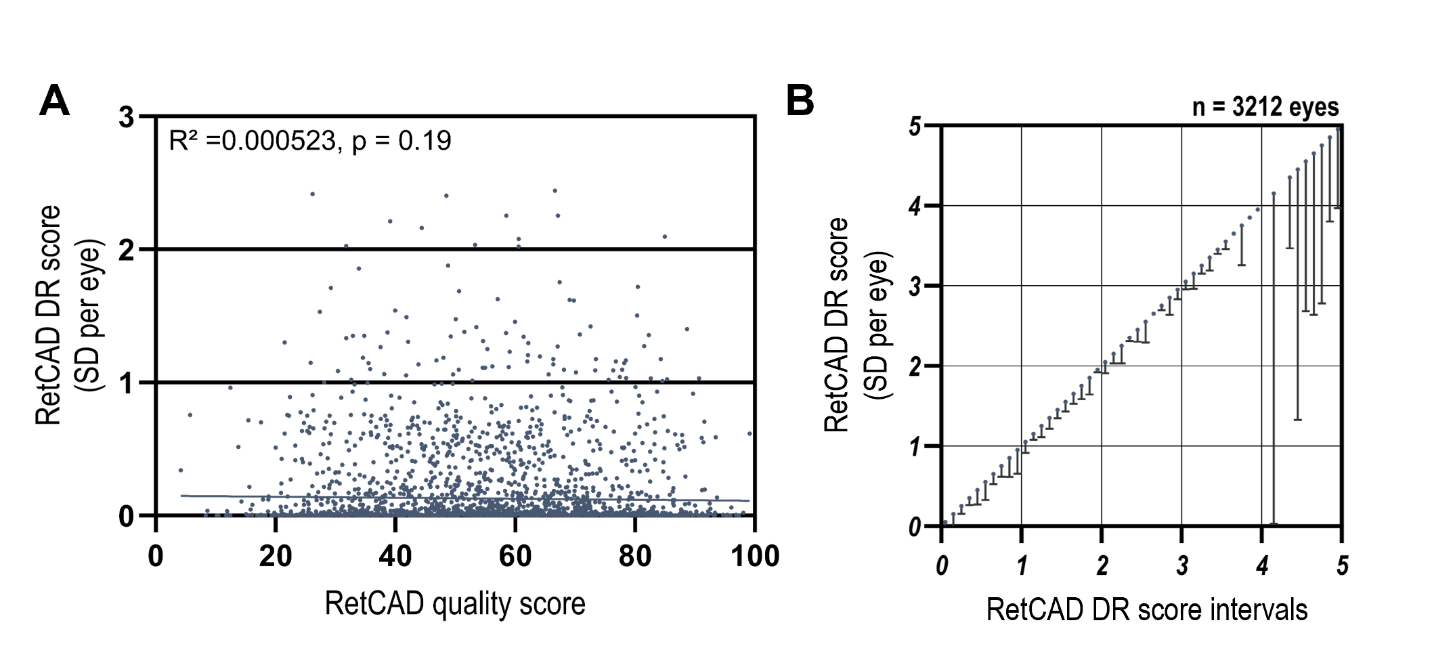
In addition, the standard deviation of the DR score was examined as a function of the DR score itself. The aim was to investigate whether higher DR scores are associated with greater variability, which could indicate that more images are required at higher DR scores to ensure reliable classification. We analyzed the standard deviation per eye (2 to 7 images) in 0.1 DR score intervals from a DR score starting at 0 to 5. The analysis reveals that the standard deviation of the DR score remains relatively stable up to DR score values of 3.5. Beyond this point, larger fluctuations in standard deviation were observed (**eFigure 4 B**).

**eFigure 4: Relationship Between RetCAD Quality Score and DR Score Standard Deviation
A:** This scatter plot illustrates the relationship between the RetCAD quality score and the standard deviation of the RetCAD DR score for 3,212 eyes. The horizontal axis represents the RetCAD quality score (ranging from 0 to 100), while the vertical axis shows the standard deviation of the DR score for each eye. The plot shows no significant correlation between the two variables (R² = 0.000523, p = 0.19), indicating that image quality, as measured by the RetCAD score, does not significantly affect the variability of DR score measurements.
**B**: The relationship between DR scores and their corresponding standard deviation for each 0.1-steps is presented to demonstrate the deviation of DR score if the DR score increases.
